# 5 days of time-restricted feeding increases fat oxidation rate but not affect postprandial lipemia: a crossover trial

**DOI:** 10.1038/s41598-022-13387-8

**Published:** 2022-06-03

**Authors:** Chih-Hui Chiu, Che-Hsiu Chen, Min-Huan Wu, Pei-Tzu Lan, Yu-Chen Hsieh, Zong-Yan Lin, Bo-Wei Chen

**Affiliations:** 1 Graduate Program in Department of Exercise Health Science, National Taiwan University of Sport, No. 16, Sec. 1, Shuang-Shih Rd., Taichung, 404 Taiwan; 2 Department of Sport Performance, National Taiwan University of Sport, Taichung, 404 Taiwan; 3 Senior Wellness and Sport Science, Tunghai University, Taichung, 404 Taiwan; 4 Clinical Trial Center, China Medical University Hospital, Taichung, 404 Taiwan; 5 Graduate Program in Department of Exercise Health Science, National Taiwan University of Sport, Taichung, 404 Taiwan

**Keywords:** Fat metabolism, Risk factors

## Abstract

Studies have revealed that time-restricted feeding affects the fat oxidation rate; however, its effects on the fat oxidation rate and hyperlipidemia following high-fat meals are unclear. This study investigated the effects of 5-day time-restricted feeding on the fat oxidation rate and postprandial lipemia following high fat meals. In this random crossover experimental study, eight healthy male adults were included each in the 5-day time-restricted feeding trial and the control trial. The meals of the time-restricted feeding trial were provided at 12:00, 16:00, and 20:00. The meals of the control trial were provided at 08:00, 14:00, and 20:00. The contents of the meals of both trials were the same, and the calories of the meals met the 24-h energy requirement of the participants. After 5 days of the intervention, the participants consumed high-fat meals on the sixth day, and their physiological changes were determined. The fasting fat oxidation rate (*p* < 0.001) and postprandial fat oxidation rate (*p* = 0.019) of the time-restricted feeding trial were significantly higher than those of the control trial. The 24-h energy consumption and postprandial triglyceride, blood glucose, insulin, glycerol, and free fatty acid concentrations of the two trials showed no significant differences (*p* > 0.05). The results revealed that 5 days of time-restricted feeding effectively increased the fasting and postprandial fat oxidation rate, but it did not affect postprandial lipemia.

## Introduction

Consuming high-fat meals increases the triglyceride (TG) level in blood plasma. Studies have discovered that large increases in postprandial TG concentration lead to high risks of cardiovascular diseases and metabolic syndrome^1^. Compared with the fasting TG concentration, the postprandial TG concentration is a more precise predictor of the risks of cardiovascular diseases and metabolic syndrome^2^. Consuming high-fat meals increases the levels of biochemical substances in blood plasma, such as the elevation of TG, free fatty acids, and remnant cholesterol. Studies have reported that these biochemical substances are major risk factors for metabolic syndrome, atherosclerosis, myocardial infarction, and coronary heart disease, all of which are associated with high mortality^3,4^. The high TG level after the consumption of high-fat meals can last for 6–8 h. As three meals daily are typically consumed by the general population^5,6^, high levels of TG may be constantly occurring in the body. Therefore, investigating methods to reduce the high TG level after eating high-fat meals is crucial for reducing the development of metabolic syndrome.

The time-restricted feeding may decrease the body weight as well as increase the fat oxidation. Studies have demonstrated that time-restricted feeding with a duration of a few weeks effectively reduced the body weight and improved metabolism^7,8^. For the short-term intervention, the 4 days of early time-restricted feeding effectively increased the fat oxidation rate^5^ and improved the 24-h blood glucose balance^6^. Recent studies have shown that increasing the fat oxidation rate after eating high-fat meals is crucial for reducing the postprandial TG level^9,10^; however, the results have remained inconsistent. Some studies have described that performing high-intensity interval training is positively correlated with decreases in the postprandial TG level^11^, whereas other studies have discovered that the increased fat oxidation rate following high-fat meals did not affect the postprandial TG level^12^. The energy expenditure during exercise or during the life^13^ may be the probable cause of discrepancies between studies. In addition, the fat oxidation which increase by time-restricted feeding whether influences postprandial TG level is yet to be determined.

Studies have discovered that time-restricted feeding can improve insulin sensitivity without weight loss^14^, increase the fat oxidation rate^15^, and decrease the fasting TG level^16^. However, whether time-restricted feeding can exert health benefits in terms of effectively reducing the increase in the TG level following high-fat meals remains unclear. The purpose of this study was to investigated the effects of 5-day time-restricted feeding on the fat oxidation rate and postprandial lipemia after the consumption of high-fat meals. Our hypothesis is that time-restricted feeding may higher the fat oxidation rate and decrease the postprandial TG concentration after a high fat meal.

## Methods

### Participants

This study recruited eight healthy male adults as research participants (age 22 ± 1.3 year, BMI 26.0 ± 0.38 kg/m^2^). All the participants had not undergone physical training; they did not exercise regularly; and they did not have any diseases that would prevent them from performing exercises, such as high blood pressure, hyperlipidemia, heart disease, joint disease, and osteoporosis. All the participants fully understood the experimental process before experiment initiation and were notified of the possible risks; they agreed to the terms of the experiment and provided their written consent. All the participants fully understood the experimental process before experiment initiation and were notified of the possible risks; they agreed to the terms of the experiment and provided their written informed consent. The participants also be informed of avoid trying to lose weight or change the dietary habit during the study. A similar number of participants and a similar recruitment method have been employed by this research team in the past. This study was approved by the Institutional Review Board of Jen-Ai Hospital (110-10) in Taiwan and registered in the ClinicalTrials.gov (Date: 22/02/2022; ID “NCT05251103”; https://register.clinicaltrials.gov). This study follows the principles of the Declaration of Helsinki and follows the recommendations proposed by the CONSORT Statement.

### Design

This study used a crossover design for the experiment. The participants were divided into the time-restricted feeding trial (abbreviated as TRF) and the control trial (abbreviated as CON). All participants consumed the same meals for 5 days. They also be informed of avoid trying to lose weight or change the dietary habit during the study. The TRF trial used the 16:8 methods to practice intermittent fasting^17^. The meals were provided at 12:00, 16:00, and 20:00. The meals of the CON trial were provided at 08:00, 14:00, and 20:00, but the consumption time was not limited. On the morning of the sixth day, all participants returned to the laboratory to consume a high-fat meal, and were investigated the TG blood levels after the meal. The participants were randomly assigned to different arms of the study to receive different treatments, and an interval of at least 14 days was maintained between the tests to avoid any effects of the preceding test on the succeeding test. Studies have reported that 4 days of intermittent fasting effectively increased the fat oxidation rate and reduced blood glucose^5,6^. Therefore, 5 days of time-restricted feeding should provide sufficient intervention time to stimulate fat oxidation rate changes. The primary outcome measure was fat oxidation rate and the blood biochemical analysis was the second.

### Protocol

#### Pretest

The pretest was to assess the total daily energy expenditure by indirect calorimetry through a series of resting assessments and exercising assessments. In addition, the gas analyzers (Vmax Series 29C, Sensor Medics, CA, USA) were used to assess the energy consumption of the participants while they were resting and performing nonmaximal intensity exercises for precisely calculating the daily calorie consumption of each participant. In the laboratory, each participant underwent heart rate monitoring with a heart rate monitor (Polar, Finland). And the energy consumption was examined by using the gas analyzers. The participants were instructed to rest quietly for 20 min in the supine position for recording their resting heart rate and energy consumption. After resting, they were required to perform nonmaximal intensity exercises for measuring their energy consumption during low-intensity activities. First, the energy consumption during standing was recorded by standing on a treadmill with a slope of 0° for 10 min. Second, the participants were instructed to walk or run at five respectively speeds, which were set as 1, 2, 3, 4, and 5 miles per hour. Each speed had been maintained for 3 min to measure the relationship between the energy consumption and heart rate of the participants during low-intensity activities.

After the indirect calorimetry assessments, the participants were asked to wear the heart rate monitor for 24 h to estimate their heart rates by daily activities performed in ordinary. The regression of heart rate and energy consumption calculated in the indirect calorimetry assessments had been used to calculate the total energy consumption of the participants. Food energy were adjusted to meet the 24-h total calories of each participant. The method recorded the energy consumption and the brand of heart rate monitors (Polar) in this study had been described elsewhere^10,18,19^.

### Formal experiment

The experiment was conducted on a 6-day period. On the first day, the participants arrived at the laboratory at 08:00 and were instructed to rest quietly for 20 min in the supine position. At the same time, gas analyzers were used to record their energy consumption. Subsequently, the participants were randomly allocated to the TRF or the CON trial. The meals of the TRF trial were provided at 12:00, 16:00, and 20:00. The participants in the TRF trial were required to consume all the food in the laboratory. On the other hand, the similar meals of the CON trial were provided at 08:00, 14:00, and 20:00. The participants in the CON trial were only required to consume the breakfast in the laboratory at 08:00 but the other meals were not limited. Except the breakfast, we reminded them to finish the meal on time by telephone. In addition to regular meals, a snack with approximately 200 cal was provided as well. The participants in the TRF were only allowed to consume the snack from 12:00 to 20:00, whereas no restrictions were imposed on the CON. The meals were provided by the investigator three times a day throughout the 6-day period and designed by the professional dieticians. The calories of each meal met the daily energy requirement of each participant, which based on the results from the pretest. The participants were instructed to maintain their habitual sleep and refrained from caffeine and exercise. The macronutrient consumption for TRF and CON were listed in Table 1.

**Table 1:** The macronutrient consumption for TRF and CON.

	TRF	CON	*p* value
Carbohydrate (g)	340.4 ± 28.0	342.2 ± 31.4	0.904
Protein (g)	124.7 ± 11.3	128.7 ± 31.4	0.599
Fat (g)	74.9 ± 7.9	72.1 ± 10.2	0.552
Total calorie (Kcal)	2533.6 ± 240.9	2532.0 ± 272.0	0.983

Values are mean SD, n = 8. *TRF* time-restricted feeding trial, *CON* control trial.

After experiment completion on the fifth day, the participants returned to the laboratory on the sixth day from 08:00 to 09:00. They rested for 10 min in the supine position, and gas analyzers were used to collect the gas data of the participants for 20 min. The average data from 5 to 15 min were used to assessed the fasting fat and carbohydrate oxidation data to avoid any error when move the equipment. Next, a catheter was inserted into the forearm of each participant to collect fasting blood samples. After blood sample collection, the participants were provided with a specific high-fat meal. The participants rested quietly in the laboratory for 4 h, and their blood lipid changes during this period were observed.

### Oral fat tolerance test (OFTT)

All oral fat tolerance test (OFTT) meals were designed and provided by dieticians, as previously described^10,20,21^. The meals included toast, butter, cheese, muesli, and cream. For every kg of the body weight of the participant, the meal provided 1.2 g of fat, 1.1 g of carbohydrate, 0.33 g of protein, and 16.5 kcal of energy. The nutritional information was obtained from the nutritional facts on food packages. During the experiment, the participants were required to consume the OFTT meal within 15 min. The average caloric and fat intake of the OFTT were 1244.1 ± 288.8 kcal and 90.5 ± 21.0 g, respectively.

### Blood collection

In the experiment, a catheter (Venflon 20G, Sweden) was inserted into the vein of the forearm, and a three-way stopcock (Connecta Ltd., Sweden) was used to collect 10 mL of blood each time. Blood was collected before meals, 30 min after meals, and every hour after meals up to the fourth hour. After each session of blood collection, 10 mL of isotonic saline water was used to clean the catheter to avoid blood clotting in the catheter.

The collected blood was immediately placed in blood collection tubes containing ethylenediaminetetraacetic acid. A cell counter was used to analyze the hematocrit (Sysmax KX-21N, Kobe, Japan). After the analysis, the blood was centrifuged for 20 min at 500×*g* at 4 °C. Blood plasma was obtained and was immediately placed in a − 80 °C refrigerator for preservation and future biochemical analysis.

### Blood biochemical analysis

The plasma were analyzed by using an automated biochemical analyzer (7020, Hitachi, Japan) with commercial reagents of TG (Wako, Osaka, Japan), glucose (GOD-PAP, Randox, Ireland), free fatty acid (Wako, Neuss, Germany) and glycerol (Randox, Antrim, Ireland). The insulin concentration in blood plasma was analyzed using a chemiluminescence immunoassay analyzer (Elecsys 2010, Roche Diagnostics, Basel, Switzerland) and commercial reagents (Roche Diagnostics, Basel, Switzerland). The intra-assay coefficients of variation of the plasma measurement were TG: 4.9%; glucose: 2.2%; free fatty acid: 2.6%; glycerol: 6.4%; and insulin: 2.6%. The fat and carbohydrate oxidation rates were calculated using the following formula^22^:

$$ {\text{Fat}}\,{\text{oxidation }}\left( {{\text{g}}/{\text{min}}} \right) \, = { 1}.{695 } \times {\text{ VO}}_{{2}} \, - { 1}.{7}0{1 } \times {\text{ VCO}}_{{2}} , $$

$$ {\text{Carbohydrate}}\,{\text{oxidation }}\left( {{\text{g}}/{\text{min}}} \right) \, = { 4}.{585 } \times {\text{ VCO}}_{{2}} \, - { 3}.{226 } \times {\text{ VO}}_{{2}} . $$

### Statistical analysis

All the data in this study are presented as average ± standard deviation. The area under the curve (AUC) of plasma parameters and substrate oxidation rates were calculated using the trapezoidal rule^23^ with excel (Microsoft, Washington, USA). The normality of the data was tested using the Shapiro–Wilk test. The fasting fat oxidation rate, blood biochemical values, and areas under the fat oxidation rate curve and the TG curve were analyzed using the paired sample t test. The postprandial fat oxidation rate and blood biochemical values were analyzed using two-way ANOVA with repeated measures. If the data were significant, the Bonferroni method was used to perform post hoc comparisons. Using G*power 3 software^24^, to achieve an alpha value of 5% and a power of 0.8, the sample size of eight was considered sufficient for this study. All other analyses were calculated by SPSS statistical software (SPSS version 20, Chicago, USA). The significance level was set at α < 0.05.

### Ethics approval and consent to participate

This study was approved by the Institutional Review Board of Jen-Ai Hospital (110-10) in Taiwan.

## Result

### Fasting plasma concentrations

The fasting fat oxidation rate of the time-restricted feeding trial was significantly higher than that of the control trial (*p* < 0.001). The 24-h energy expenditure, resting energy expenditure, fasting carbohydrate and the concentrations of TG, blood glucose, insulin, free fatty acids, and glycerol between the two trials were not significantly different (*p* > 0.05) (Table 2).

**Table 2:** The participants physiological information and fasting plasma biochemistry.

	TRF	CON	*p* value
24 h energy expenditure (Kcal)	2541.5 ± 1406.2	2388.6 ± 1370.4	0.419
Resting energy expenditure (Kcal)	1524.6 ± 249.6	1506.4 ± 455.0	0.886
TG (mmol/L)	1.16 ± 0.4	1.45 ± 0.7	0.077
Glucose (mmol/L)	70.6 ± 5.3	71.0 ± 3.2	0.793
Insulin (pmol/L)	7.8 ± 3.2	7.6 ± 4.5	0.931
NEFA (mmol/L)	0.38 ± 017	0.35 ± 0.08	0.655
Glycerol (μmol/L)	33.4 ± 17.0	27.9 ± 10.5	0.190
Fat oxidation (g/min)	0.14 ± 0.05	0.04 ± 0.03	< 0.001
Carbohydrate oxidation (g/min)	0.21 ± 0.12	0.26 ± 0.14	0.167

Values are mean SD, n = 8. *TRF* time-restricted feeding trial, *CON* control trial, *TG* triglyceride, *NEFA* non-esterified fatty acids.

### Postprandial fat oxidation rate and area under the curve

**Figure 1:**
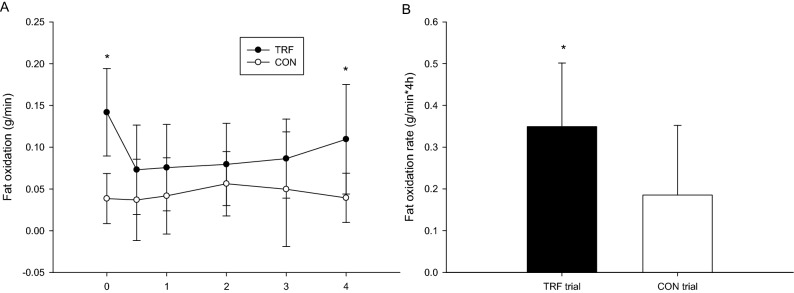
The postprandial fat oxidation over the 4 h (**A**) and the fat oxidation rate area under the curve in 4 h (**B**). *TRF was significantly higher than those for the CON.

The postprandial fat oxidation rate of the TRF trial was significantly higher than that of the CON trial (trial × time, *p* = 0.019; trial, *p* = 0.004; time, *p* = 0.256; Fig. 1A). The AUC of postprandial fat oxidation rate in the TRF trial was significantly larger than that of the CON trial (*p* = 0.02, Fig. 1B). The effect size (Cohen's dz) was 1.03 for the fat oxidation AUC.

### Postprandial TG concentration and area under the curve

**Figure 2:**
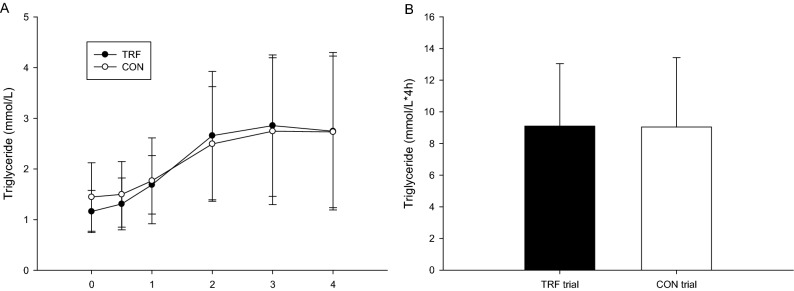
The postprandial triglycerides concentrations over the 4 h (**A**) and the TG area under the curve in 4 h (**B**). *Mean TRF was significantly higher than those for the CON.

Postprandial TG concentrations were not significantly different between the TRF and CON trials (trial × time, *p* = 0.531; trial, *p* = 0.850; time, *p* < 0.001; Fig. 2A). The AUC in the TG concentration were also not significantly different between the two trials (*p* = 0.956, Fig. 2B).

### Postprandial blood biochemical indices

**Figure 3:**
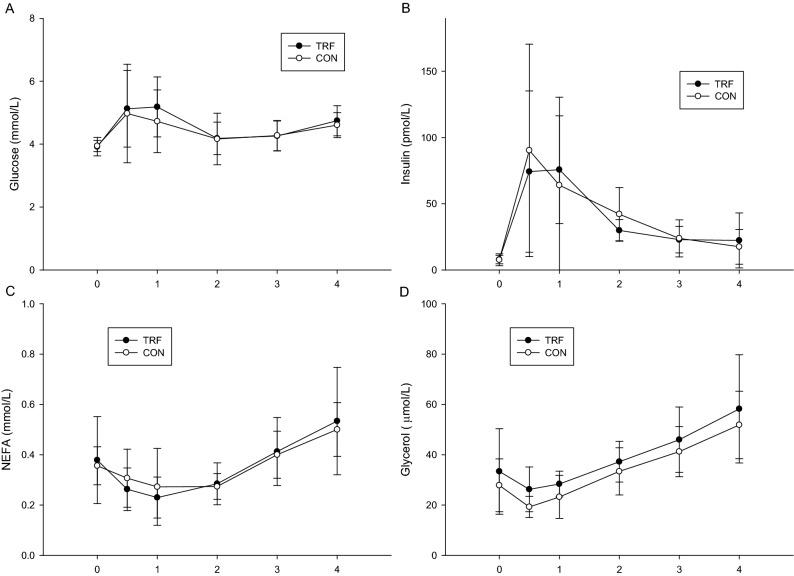
The postprandial glucose concentrations over the 4 h (**A**), insulin concentrations over the 4 h (**B**), glycerol concentrations over the 4 h (**C**) and non-esterified fatty acids concentrations over the 4 h.

The plasma glucose concentration was not significantly different between the two trials (trial × time, *p* = 0.712; trial, *p* = 0.456; time, *p* = 0.023; Fig. 3A). Figure 3B indicated that the insulin concentration was not significantly different between the trials (trial × time, *p* = 0.564; trial, *p* = 0.558; time, *p* = 0.027; Fig. 3B). Figure 3C indicated that the free fatty acid concentration was not significantly different between the trials (trial × time, *p* = 0.598; trial, *p* = 0.976; time, *p* < 0.001; Fig. 3C). Figure 3D indicated that the glycerol concentration was not significantly different between the trials (trial × time, *p* = 0.925; trial, *p* = 0.064; time, *p* < 0.001; Fig. 3D).

## Discussion

In this study, meals were provided that met the 24-h energy requirement of each participant for 5 days. The intervention was time-restricted feeding conducted at different parts of the day. The results revealed that time-restricted feeding effectively increased the fasting fat oxidation rate and the fat oxidation rate after the consumption of high-fat meals. However, the increased fat oxidation rate exerted no effects on the TG level following high-fat meals, 24-h energy consumption, resting energy expenditure, or reactions of blood biochemical substances.

This study confirmed the fasting fat oxidation rate and the fat oxidation rate after the consumption of high-fat meals were effectively increased via the 5-day of time-restricted feeding period. On the contrary, the 24-h energy expenditure and resting energy expenditure showed no influence by the restricted feeding. Studies applying time-restricted feeding have mostly used interventions with a duration of a few weeks, and the results showed that time-restricted feeding decreased body weight and improved metabolism^7,8^. Studies that utilized short-term time-restricted feeding have discovered that 4 days of early time-restricted feeding (consuming dinner before 15:00) effectively increased the fat oxidation rate and reduced appetite, however, it did not affect 24-h energy expenditure and resting energy expenditure^5^. A similar study demonstrated that 4 days of early time-restricted feeding improved the 24-h blood glucose balance^6^. In contrast to the aforementioned studies, this study used late time-restricted feeding (consuming dinner before 20:00). In addition, all the meals were prepared by the research team and were directly provided to the participants; hence, in this study, the diet of the participants could be more precisely controlled, instead of the participants consuming their own food. This study discovered that late time-restricted feeding produced results similar to those achieved by early time-restricted feeding. In addition, compared with the control trial, time-restricted feeding did not affect the 24-h energy metabolism of the time-restricted feeding trial, and time-restricted feeding effectively increased the fasting fat oxidation rate and the fat oxidation rate after the consumption of high-fat meals. However, the glycerol and free fatty acid concentrations of the two trials were not different. Therefore, the exact mechanism through which time-restricted feeding increased the fat oxidation rate was unknown.

In this study, time-restricted feeding could effectively increase the fasting fat oxidation rate and the postprandial fat oxidation rate, but it did not affect the TG level after the consumption of high-fat meals. This result indicated that 5 days of short-term time-restricted feeding resulted in a shorter action time for the higher fat oxidation rate, which may not effect on the postprandial TG level. The possible mechanisms may be due to the increased of adrenergic activity^25^ or the thermic effect of food^5^. Chiu et al. used three high-fat meals per day to change the fat oxidation rate of participants; although this method effectively increased the fat oxidation rate, it did not affect the TG level after the consumption of high-fat meals^12^. This study demonstrated that the fat oxidation rate of the time-restricted feeding trial was significantly higher than that of the control trial; however, glycerol and free fatty acid concentrations were not significantly different. Therefore, although short-term time-restricted feeding effectively increased the fat oxidation rate, it did not affect the postprandial TG reaction.

Another possible reason for the intervention not affecting the TG level after the consumption of high-fat meals is that 5-day time-restricted feeding did not affect blood glucose and insulin concentrations. Studies have suggested that insulin sensitivity is a major factor that affects the TG level after the consumption of high-fat meals^26^. Compared with late time-restricted feeding, early time-restricted feeding reduced postprandial blood glucose concentration to a higher extent in a previous study^27^. However, that study did not limit the calorie intake, and participants were 55 years old and were at a high risk of diabetes. In comparison, this study provided all the meals to the participants during the experiment to ensure that the calorie intake of all the participants was equal. In addition, this study controlled the calorie intake to ensure that it met the 24-h energy requirement of the participants, and the results revealed that fasting and postprandial blood glucose concentrations and the insulin concentration were unaffected. Accordingly, the insulin sensitivity of the participants remained unchanged; thus, the postprandial TG level was unaffected.

The male subjects recruited in this study belonged to healthy population, which had the low fasting TG levels. However, it is not certain in the results would apply to overweight, middleaged and older adults, or in at-risk populations. The fasting fat oxidation rate were 0.14 g/min in the TRF trial. It was 30–50% higher than our previous studies^12^. Therefore, the 5 days of time-restricted feeding not only increased the fat oxidation rate in healthy normal weight male subjects as overweight subjects^5^, but also maximized the fat oxidation rate. This may be an explanation that why the fat oxidation cannot be further increased after consuming a high fat meal. Nonetheless, this present study indicated that time-restricted feeding increased the fasting and postprandial fat oxidation, which likely lead to improved fat metabolism or cardiometabolic health^28^. Moreover, the further research is required to investigate the effect of TRF on postprandial response after a high fat meal in the overweight or at-risk populations.

The main of this study was the calculation of 24-h energy consumption. The 24-h energy consumption was determined through calculation, rather than through measurement by methods such as those using the respiratory chamber. Calculations would not be as accurate as actual measurements. Studies have tested 24-h energy consumption and yielded robust results using methods similar to that used in the present study^10,18,19^. Therefore, we believe that this method is still credible. The other limitation was that we only measure the 4th hour postprandial outcomes. Further study may be needed to investigate the postprandial outcomes for a longer time.

## Conclusion

This study discovered that consuming meals with the same amount of calories for 5 days and using time-restricted feeding as the intervention can effectively increase the fasting fat oxidation rate and the fat oxidation rate after the consumption of high-fat meals. However, the increased fat oxidation rate did not increase the TG level after the consumption of high-fat meals in the healthy male participants. The further research is required to investigate the effect of time-restricted feeding on postprandial response after a high fat meal in the overweight or at-risk populations.

## Data Availability

All relevant materials are presented in the present manuscript.

## References

[1] Liu H-H, Li J-J (2015). Aging and dyslipidemia: A review of potential mechanisms. Ageing Res. Rev..

[2] Nordestgaard BG, Benn M, Schnohr P, Tybjærg-Hansen A (2007). Nonfasting triglycerides and risk of myocardial infarction, ischemic heart disease, and death in men and women. JAMA J. Am. Med. Assoc..

[3] Bansal S (2007). Fasting compared with nonfasting triglycerides and risk of cardiovascular events in women. JAMA J. Am. Med. Assoc..

[4] Langsted A (2011). Nonfasting cholesterol and triglycerides and association with risk of myocardial infarction and total mortality: The Copenhagen City Heart Study with 31 years of follow-up. J. Intern. Med..

[5] Ravussin E, Beyl RA, Poggiogalle E, Hsia DS, Peterson CM (2019). Early time-restricted feeding reduces appetite and increases fat oxidation but does not affect energy expenditure in humans. Obesity.

[6] Jamshed H (2019). Early time-restricted feeding improves 24-hour glucose levels and affects markers of the circadian clock, aging, and autophagy in humans. Nutrients.

[7] Pellegrini M (2020). Effects of time-restricted feeding on body weight and metabolism. A systematic review and meta-analysis. Rev. Endocr. Metab. Disord..

[8] Gabel K (2018). Effects of 8-hour time restricted feeding on body weight and metabolic disease risk factors in obese adults: A pilot study. Nutr. Healthy Aging.

[9] Trombold JR, Christmas KM, Machin DR, Kim I-Y, Coyle EF (2013). Acute high-intensity endurance exercise is more effective than moderate-intensity exercise for attenuation of postprandial triglyceride elevation. J. Appl. Physiol..

[10] Yang T-J, Wu C-L, Chiu C-H (2018). High-intensity intermittent exercise increases fat oxidation rate and reduces postprandial triglyceride concentrations. Nutrients.

[11] Wilhelmsen A (2019). Chronic effects of high-intensity interval training on postprandial lipemia in healthy men. J. Appl. Physiol..

[12] Chiu C-H, Yang T-J, Chen C-H, Zeng M-J (2019). High fat meals increases postprandial fat oxidation rate but not postprandial lipemia. Lipids Health Dis..

[13] Chiu C-H, Chen C-H, Wu M-H, Ding Y-F (2020). Nonexercise activity thermogenesis-induced energy shortage improves postprandial lipemia and fat oxidation. Life.

[14] Sutton EF (2018). Early time-restricted feeding improves insulin sensitivity, blood pressure, and oxidative stress even without weight loss in men with prediabetes. Cell Metab..

[15] Liu B, Page AJ, Hutchison AT, Wittert GA, Heilbronn LK (2019). Intermittent fasting increases energy expenditure and promotes adipose tissue browning in mice. Nutrition.

[16] Liu B (2019). Intermittent fasting improves glucose tolerance and promotes adipose tissue remodeling in male mice fed a high-fat diet. Endocrinology.

[17] Moro T (2016). Effects of eight weeks of time-restricted feeding (16/8) on basal metabolism, maximal strength, body composition, inflammation, and cardiovascular risk factors in resistance-trained males. J. Transl. Med..

[18] Silva AM (2015). Accuracy of a combined heart rate and motion sensor for assessing energy expenditure in free-living adults during a double-blind crossover caffeine trial using doubly labeled water as the reference method. Eur. J. Clin. Nutr..

[19] Santos DA (2014). Validity of a combined heart rate and motion sensor for the measurement of free-living energy expenditure in very active individuals. J. Sci. Med. Sport.

[20] Chiu C-H (2014). Energy replacement using glucose does not increase postprandial lipemia after moderate intensity exercise. Lipids Health Dis..

[21] Chiu C-H, Yang T-J, Liang HJ, Chang C-K, Wu C-L (2018). A single bout of exercise reduces postprandial lipemia but has no delayed effect on hemorheological variables. Chin. J. Physiol..

[22] Frayn K (1983). Calculation of substrate oxidation rates in vivo from gaseous exchange. J. Appl. Physiol..

[23] Matthews J, Altman DG, Campbell M, Royston P (1990). Analysis of serial measurements in medical research. BMJ.

[24] Faul F, Erdfelder E, Lang A-G, Buchner A (2007). G* Power 3: A flexible statistical power analysis program for the social, behavioral, and biomedical sciences. Behav. Res. Methods.

[25] Jensen MD, Haymond MW, Gerich JE, Cryer PE, Miles JM (1987). Lipolysis during fasting. Decreased suppression by insulin and increased stimulation by epinephrine. J. Clin. Investig..

[26] Guerci B (2000). Relationship between altered postprandial lipemia and insulin resistance in normolipidemic and normoglucose tolerant obese patients. Int. J. Obes..

[27] Hutchison AT (2019). Time-restricted feeding improves glucose tolerance in men at risk for type 2 diabetes: A randomized crossover trial. Obesity.

[28] Wolfe AS, Burton HM, Vardarli E, Coyle EF (2020). Hourly 4-s Sprints Prevent Impairment of Postprandial Fat Metabolism from Inactivity. Med. Sci. Sports Exerc..

